# Should They Be Relinquished for the Countryside?

**Published:** 1920-06-26

**Authors:** 


### THE LONDON HOSPITAL SITES.

### Should they be Relinquished for the Countryside?

A paragraph appeared in *The Times* on Saturday last in which reference was made to the suggestion that the big London hospitals might profitably dispose of their present valuable sites in the City, maintain smaller establishments in London, and re-establish the larger hospitals further out. Secretaries of some of the larger London hospitals who might be affected by such a revolutionary proposal if it were carried into effect seemed reluctant to express any decided opinions on the matter when invited to do so by a representative of The Hospital. One gathered, however, from conversation with several of them that the general attitude of the profession on first consideration is rather against the proposal.

The idea, of course, is not new. It has its attractive side, especially just now, when most of the big voluntary hospitals are working under severe financial embarrassments. The revenue to be derived from the disposal of some of the present sites would run into millions, and would provide ample funds for the erection of much more elaborate and up-to-date institutions in the country. What is sometimes forgotten, however, by people who advocate these reforms is that a hospital exists primarily for the benefit and convenience of its patients. The secretary of St. Thomas's, who discussed the matter at some length, did not overlook this aspect of the question. Though he strongly advocated the establishment of auxiliary hospitals in the country where out-patients who simply needed rest and quiet to prevent a more serious illness, and semi-chronic cases, could go and recuperate, he insisted on the retention of the bigger hospitals near the centre of population.

Besides this natural antipathy of the patients to being sent away to a strange district, there are other great difficulties in the way. The secretary of St. Thomas's indicated some of these in explaining the relationship of the out-patients' to the in-patients' department. The former, of course, would still have to remain near the centre of population, but it would be very difficult to separate the two. The out-patients' department, he claimed, was actually quite as important a part of a big general hospital as the in-patients' department, and the same individuals in many cases worked in both. Then in a big hospital were a number of specialised departments, such as *x*-ray departments for investigation purposes, bacteriological and chemical laboratories, Swedish remedial exercises for treatment of cases, and large and expensive electrical departments. All these, he said, would have to remain with the big hospital taking in the cases near the centre of population.

"You cannot take away all your big hospitals from London," he added. "London is the feeding-ground. But thoroughly efficient out-patients' departments, such as I want to see attached to St. Thomas's, could safely feed auxiliary hospitals in the country with patients who merely need rest and quiet and the large number of cases of a semi-chronic kind who mostly need a long rest in bed and very little treatment. It would be a magnificent thing if each of the big London hospitals could have attached to them country hospitals, somewhat in the nature of preventive convalescent homes, to which both these classes of cases could be sent."

The idea of removing St. Bartholomew's Hospital from its present site was seriously considered in 1903, when a Mansion House inquiry was held, presided over by the Lord Mayor. A committee of seventeen well-known public men at that time resolved, with only one dissentient, that it was "impossible in the public interest to entertain the idea of removing St. Bartholomew's Hospital from its present site." The present secretary is still of that opinion. The proposal, if put into execution, he thinks, would alter the whole nature of hospital work in regard to staffing, etc. It would be disastrous, in his opinion, to the medical schools.

The London Hospital, though it still retains its big establishment in the East End, has already developed to a certain extent along the lines of decentralisation. It has a home at Reigate which is used as an annexe, and not merely as a convalescent home. The really bad cases, of course, do not go there, but patients are taken down in ambulances and receive a certain amount of nursing after the actual skilled treatment by the specialist has been done in London. "I think that is doubtless the line of hospital development for the future," the secretary said. "You can never entirely abolish the big teaching hospitals in the big centres. I do not think that would be possible. You cannot put all the material on which the students are to be taught down in the country. The specialists who teach the students have to live in London. So, from this point of view of the teaching hospital, you cannot go to the extent of selling the site and leaving a few beds here for casualties."

[We shall make further reference to this subject next week.]

